# A Surgical Opinion in a 36-Week Pregnant with Tibia Fracture: Intramedullary Nailing

**DOI:** 10.1155/2016/1454835

**Published:** 2016-12-13

**Authors:** Celal Bozkurt, Baran Sarikaya

**Affiliations:** Department of Orthopaedics and Traumatology, Faculty of Medicine, Harran University, Sanliurfa, Turkey

## Abstract

The operative treatment of tibial fractures in late pregnancy is a controversial issue that is rarely discussed in the literature. Here we present a case of a tibial diaphyseal fracture in a woman that was 36 weeks pregnant, which was treated with intramedullary nails under noninvasive foetal monitoring with cardiotocography. The patient underwent a successful surgery, and no harm or adverse events to either the mother or the foetus were reported during or after the procedure. Following surgery, the mother had a comfortable pregnancy and a normal spontaneous vaginal delivery with a healthy newborn.

## 1. Introduction

A fracture of the tibial shaft is one of the most common long bone fractures in the body [1]. Tibial diaphyseal fractures can be treated either surgically or conservatively; however, surgical treatment establishes a better union and rapid resumption of full weight-bearing activities [2, 3]. Conservative treatment consists of a closed reduction and an above-the-knee cast for at least two months. The patients are mobilized with crutches and are not allowed to bear weight during this period of time. Among the surgical treatment options, the three most common methods for fixation are plating, intramedullary nailing, and external fixation. Of these options, the literature suggests that intramedullary nails are most commonly indicated for midshaft fractures, while external fixation is generally indicated for damage control with open fractures or compromised soft tissues [4].

During the postoperative plating period, the patients bear partial weight until a union is achieved. External fixator may impede the natural delivery process [4]. After tibial nailing, weight-bearing mobilization in the early postoperative period is possible. In addition to a better fracture union, it is more comfortable and has less of an effect on the delivery choice.

After conservative treatment and tibial plating for at least for two months, the patients are mobilized as non-weight-bearing (plaster) or partial weight-bearing (plating) through crutches. However, this period is uncomfortable for the mother both before and after the delivery while caring for a baby, and there is an increased risk for deep venous thrombosis (DVT) due to decreased mobilization. Moreover, pregnancy designates a hypercoagulable status, and unfractionated heparin and low molecular weight heparin (LMWH) can be used for prophylaxis [5]. Because of the cast, vaginal delivery is difficult for the mother, which can affect the method of delivery [6].

Orthopaedic emergencies should be treated as such, regardless of the pregnancy status of the patient [7]. Closed extremity fractures can be managed nonoperatively or be delayed until postpartum, when appropriate [8]. However, an accelerated fracture union during pregnancy [9] will complicate a postponed surgery. In pregnancy, there is an increase in the level of steroid hormones, initially with progesterone in the first trimester, followed by the oestrogens and prolactin in the second and third trimesters. Oestrogen has well-documented effects on bone formation and remodelling during fracture healing [10, 11]. Other controversial issues are the anaesthetic agents used [12], radiation exposure [13], and probable thromboembolic events due to immobilization. On the basis of these adverse events, surgical management is a difficult decision for both the surgeon and the mother. Unfortunately, there are limited reports on the management of long bone fractures in late pregnancy.

Here we report the case of a patient with a tibial diaphyseal fracture at 36 weeks of gestation treated with tibial nailing.

## 2. Case Report

A 36-year-old pregnant woman presented to emergency department following an accidental fall down the stairs. She was evaluated by both an orthopaedic surgeon and an obstetrician. There was no history of any medical disorder, and she had had an uneventful pregnancy until this accident.

**Figure 1:**
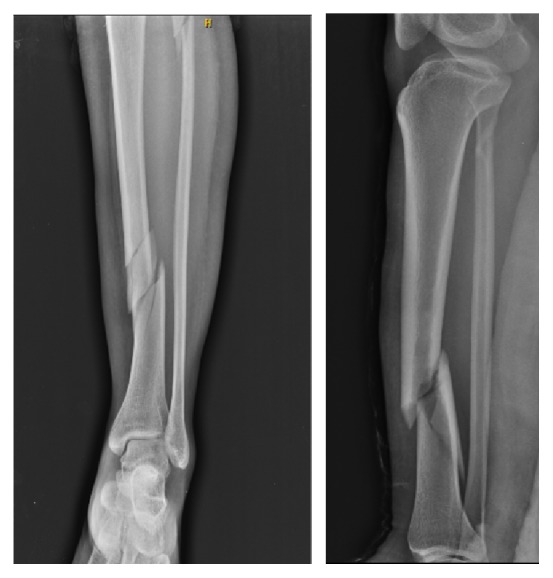
Radiographs showing the anteroposterior and lateral views of tibial shaft fracture.

Upon examination, there was no evidence of a neurovascular deficit or compartment syndrome, and there were no open wounds. Radiographic imaging showed a left tibia and fibula diaphyseal fracture classified as 42-B1 according to the Arbeitsgemeinschaft für Osteosynthesefragen (AO) (Figure 1). The fracture was stabilized with an above-the-knee splint.

Following the orthopaedic examination, the obstetrician evaluated the foetus and the mother. According to the obstetric ultrasonography and cardiotocography (CTG), no pathologies were detected. There were no restraints for surgical treatment.

The patient was brought to the operating theatre after stabilization. Spinal anaesthesia was applied, and she was positioned supine on the operating table. She was tilted 15° by placing a wedge under her right buttock to reduce the inferior vena cava pressure [5]. A lead apron was placed over the patient's abdomen to minimise the radiation dose to the foetus. After spinal anaesthesia, the foetus was monitored with continuous CTG during the surgery. An obstetrician stood by in the theatre in case of an emergency caesarean section.

**Figure 2:**
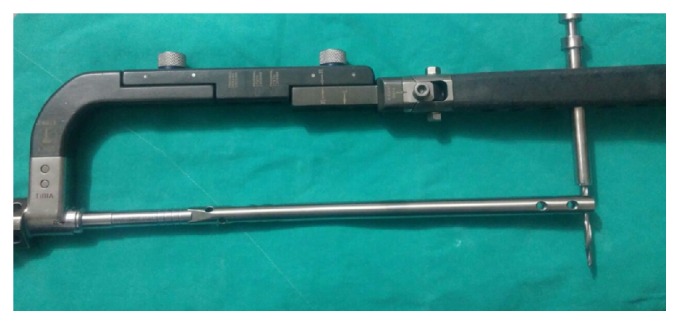
Distal guide.

The tibial nail (manufactured by Tasarımmed®, Istanbul, Turkey) was inserted via a standard infrapatellar incision. During reamerization, the foetal wellbeing was monitored with CTG, which was reactive throughout the surgery, and no adverse reactions were observed. When encountering some resistance during the reamerization, we increased the reamer diameter size by one (1 mm increase) and then stopped reaming (we did not use additional fluoroscopy to decrease the radiation exposure) to decrease the operation time. We inserted a titanium tibial nail locked with four screws (Figure 1) and used the distal guide of the nail (Figure 2) to minimise the radiation dose.

During the postoperative period, the mother and the foetus were evaluated serially by the obstetrician. The obstetric examination and CTG were normal throughout the postoperative period. This patient was administered analgesics, cold application, and 4000 IU/day subcutaneous enoxaparin for prophylaxis. On postoperative day one she was mobilized, permitting partial weight-bearing with crutches. She was discharged home on postoperative day three.

**Figure 3:**
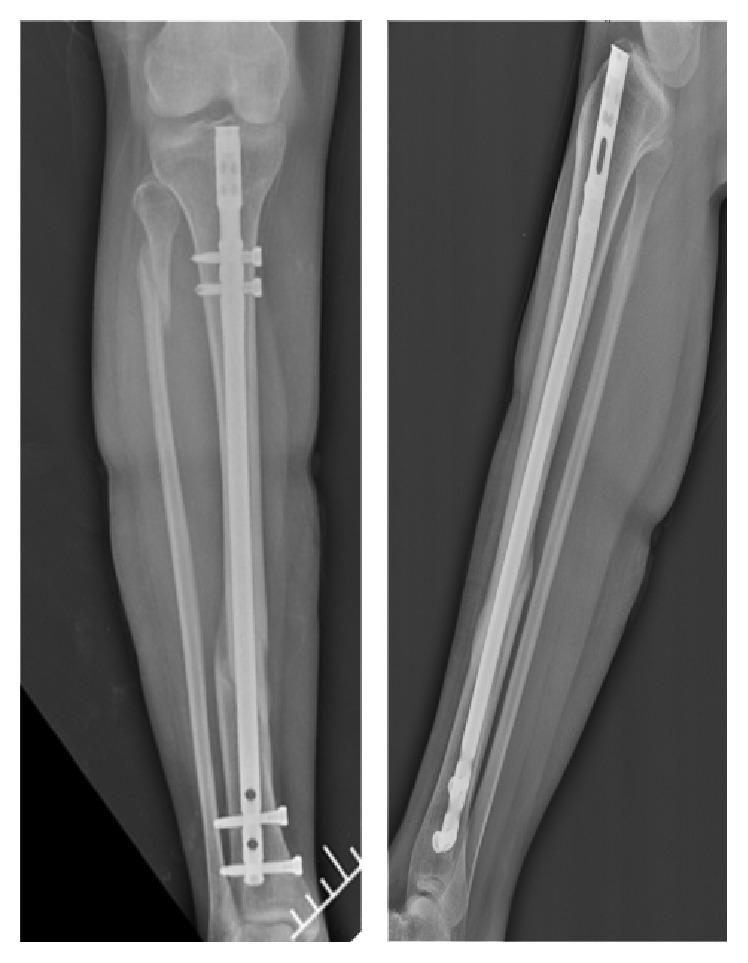
Radiographs showing united tibia at four months.

This mother had no problems during her final weeks of pregnancy. At the 40th week, she had a healthy newborn via vaginal delivery. Two days after delivery, the mother and newborn were discharged home. We saw fracture union in the fourth-month radiographs (Figure 3).

## 3. Discussion

The operative treatment of closed fractures in the third trimester of pregnancy remains controversial. The anaesthetic [12] and radiation exposure [13] to the foetus and probable embolic events [5] frighten both physicians and pregnant patients. We could find only a few cases in the literature regarding tibial fracture treatment in the third trimester of pregnancy [6, 8, 9, 14]. Since there are so few published case reports in the literature, there is no consensus on the appropriate management of maternal fractures during pregnancy.

Anaesthetics affect both the mother and the foetus; therefore, anaesthesia is more complex during pregnancy. Organogenesis occurs in the first trimester, and although there are no anaesthetic agents shown to be teratogenic, surgical treatments are usually delayed until the second trimester. From the second trimester until the end of pregnancy, the foetus will be less affected by anaesthesia [12].

Radiation exposure is another risk factor for the foetus. It affects the foetus more during the first trimester since the development of the central nervous system (CNS) is faster and more susceptible to radiation during this period. After 25 weeks of gestation, the CNS becomes more resistant to radiation; however, the cumulative radiation effects are still important. The foetus can absorb up to 100 mGy (milligray) of radiation safely, and, as long as this is not exceeded, X-rays can be used during surgery [13].

After conservative treatment for this type of fracture, the mother is non-weight-bearing, using crutches, so mobilization will be decreased. This period can be uncomfortable for the patient; an above-the-knee cast will affect the delivery, and a caesarean section should be planned [6]. After tibial nailing, there is no splint or cast, so the mother can choose vaginal delivery.

In the third trimester, a tibial fracture can be stabilized with an above-the-knee splint, and the surgery can be done following a caesarean section during the postpartum period [8]. In this situation, the mother's mobilization will be decreased, and she may not be able to choose a natural delivery [6]. In the literature, it has been reported that because of the changed hormonal status during pregnancy, the fracture union is accelerated [9]. In this case, a surgery planned later will be technically harder.

A gravid uterus can increase the pressure on the inferior vena cava, especially when the patient is in the supine position. The reduction in the preload caused by the compression of the inferior vena cava can lead to hemodynamic instability. If possible, the patient should be placed in the left lateral decubitus position. If there is any contraindication to lateral decubitus positioning, the patient should be tilted approximately 15° with a wedge under the right buttock, which displaces the uterus laterally. We chose the latter method for the surgery in this case, which was a more favourable position [5].

During tibial nailing, it has been shown that the distal locking process is responsible for at least 50% of the fluoroscopic exposure of the whole operative procedure [15, 16]. The average time for one distal locking screw is 17.9 minutes [17]. To decrease the operation time and radiation exposure, we used the distal locking system of the nail. Therefore, it took only five minutes to lock the nail distally, and we decreased the radiation exposure.

The stress on the foetus and mother may increase during surgery because of the anaesthetic exposure and surgical procedure, especially reamerization. Monitoring the foetus with CTG is important, and we did not detect any adverse activity during the surgical procedure while using CTG. Surgery and pregnancy increase the risk of DVT; however, heparin and LMWH are safe to use during pregnancy, so they can be used for prophylaxis [5, 18].

While reviewing the literature, we encountered only one case report on the surgical treatment of a tibial fracture during pregnancy [14]. The few other reports were on conservative management and the surgical treatment after a caesarean section [8]. For example, Ahmad et al. reported an accelerated tibial fracture union and no need for surgical treatment in the third trimester of pregnancy [9].

When deciding on the appropriate management for a fracture during pregnancy, the gestational age, type of the fracture, best way to obtain an acceptable fracture union, probable harm, risks to the mother and foetus, and the comfort of the mother and newborn should all be taken into consideration.

After obstetric ultrasonography, obstetric examination, and evaluation of the foetus, if the mother and foetus are stable, surgery can be planned. The surgical treatment chosen should not only be important for achieving appropriate bone alignment and union, but also for the comfort of the mother and newborn. Surgery after the delivery can negatively affect a mother's care of her baby, since she will be hospitalized and immobilized for at least few days. Moreover, a second surgery will add to the psychological stress of a mother who has already undergone a caesarean section. We believe that this approach may negatively affect mother-newborn bonding, decrease the care given by the mother to her baby, and adversely influence the emotional status of the mother.

Fracture management in a pregnant patient should be multidisciplinary, including orthopaedic surgeon, obstetrician, anaesthetist, and neonatologist. Before deciding on a treatment, one should consider not only the fracture, but also the comfort of the mother and foetus before and after delivery.
